# Synthesis and Characterization of Thiolated Nanoparticles Based on Poly (Acrylic Acid) and Algal Cell Wall Biopolymers for the Delivery of the Receptor Binding Domain from SARS-CoV-2

**DOI:** 10.3390/pharmaceutics16070891

**Published:** 2024-07-02

**Authors:** Ileana García-Silva, Susan Farfán-Castro, Sergio Rosales-Mendoza, Gabriela Palestino

**Affiliations:** 1 Biotechnology Section, Center for Research in Health Science and Biomedicine, Autonomous University of San Luis Potosí, Av. Sierra Leona 550, Lomas de San Luis, San Luis Potosí 78210, Mexico; a196037@alumnos.uaslp.mx (I.G.-S.); a205029@alumnos.uaslp.mx (S.F.-C.); 2 Biopolymers and Nanostructures Laboratory, School of Chemical Sciences, Autonomous University of San Luis Potosí, Manuel Nava 6, Av. Dr. Manuel Nava, Zona Universitaria, San Luis Potosí 78210, Mexico; 3 Recombinant Biopharmaceuticals Laboratory, School of Chemical Sciences, Autonomous University of San Luis Potosí, Manuel Nava 6, Av. Dr. Manuel Nava, Zona Universitaria, San Luis Potosí 78210, Mexico

**Keywords:** thiomer, poly (acrylic acid), mucoadhesion, antigen carrier, adjuvant

## Abstract

The COVID-19 pandemic required great efforts to develop efficient vaccines in a short period of time. However, innovative vaccines against SARS-CoV-2 virus are needed to achieve broad immune protection against variants of concern. Polymeric-based particles can lead to innovative vaccines, serving as stable, safe and immunostimulatory antigen delivery systems. In this work, polymeric-based particles called thiolated PAA/Schizo were developed. Poly (acrylic acid) (PAA) was thiolated with cysteine ethyl ester and crosslinked with a *Schizochytrium* sp. cell wall fraction under an inverse emulsion approach. Particles showed a hydrodynamic diameter of 313 ± 38 nm and negative Zeta potential. FT-IR spectra indicated the presence of coconut oil in thiolated PAA/Schizo particles, which, along with the microalgae, could contribute to their biocompatibility and bioactive properties. TGA analysis suggested strong interactions between the thiolated PAA/Schizo components. In vitro assessment revealed that thiolated particles have a higher mucoadhesiveness when compared with non-thiolated particles. Cell-based assays revealed that thiolated particles are not cytotoxic and, importantly, increase TNF-α secretion in murine dendritic cells. Moreover, immunization assays revealed that thiolated PAA/Schizo particles induced a humoral response with a more balanced IgG2a/IgG1 ratio. Therefore, thiolated PAA/Schizo particles are deemed a promising delivery system whose evaluation in vaccine prototypes is guaranteed.

## 1. Introduction

Vaccination has become one of the most valuable medical strategies in public health to prevent the negative impact of several infectious diseases [1,2]. The basis for most of the vaccines is the induction of protective humoral- or cell-mediated immune responses by administering an antigenic formulation, most of the times by injection. The COVID-19 pandemic will become a historical reference on the impact of vaccination since several vaccines were developed in record time and were used to effectively fight against the emerging pathogen. Most of the applied vaccines were based on RNA, adenoviral vectors, and recombinant proteins. The development path continues, and several groups have explored subunit vaccines based on DNA, peptides, and rationally designed recombinant proteins [3,4]. The use of peptides or recombinant proteins offers greater safety as they are less reactogenic and induce very specific immune responses directed against the protective antigens; however, their immunogenicity is often lower, caused by a low complexity, and thus adjuvants are required to obtain a sufficiently immunogenic formulation [5].

Polymeric-based particles made with natural and synthetic components stand as a highly attractive strategy to develop advantageous delivery systems since the properties of the individual components can be synergistically combined. For instance, it has been reported that synthetic polymers improve the stability of the nanostructure while natural components provide biocompatibility, low-toxicity, and bioactivity [6]. Proteins and polysaccharides obtained from different organisms such as fungi and algae have been used as the natural polymers source for such purpose. *Schizochytrium* sp., a thraustochytrid, is a heterokont marine microalga used at the industrial level for the production of high-value products; this species has been proposed as a production host and delivery vehicle for vaccines [7]. Interestingly, *Schizochytrium* sp. synthesizes several immunomodulating compounds, which could add an additional advantage when used for the design of delivery systems intended to stimulate immune system cells. Moreover, since the cell wall is usually disposed when *Schizochytrium* sp. is used at the industrial level, its application in the synthesis of added value materials is a highly convenient application for a residual biomass. From an environmental point of view, the use of eco-friendly compounds in synthesis methods is also an interesting approach in the development of biomaterials [8].

Thiomers are polymer-based biomaterials formed by the covalent addition of sulfhydryl-bearing ligands attached to a backbone of natural or synthetic polymers such as chitosan or poly (acrylic acid) (PAA) [9]. This type of material offers several advantages for the development of vehicles for drug delivery, such as permeation properties, inhibitory effects on degradative enzymes, and enhanced uptake by immune cells, which is of particular interest for vaccine development [10]. PAA is a hydrophilic, non-toxic, biocompatible, and biodegradable synthetic polymer that shows high water absorption, and it has been used to manufacture nanomaterials for biomedical applications, including biosensing, drug delivery, tissue engineering, and antimicrobial materials design [11]. Our previous report on PAA-based microparticles using a model antigen revealed their potential as antigen delivery systems [12]. Among the synthetic compound candidates for the design of hybrid particles with biopolymers stands thiolated PAA, which provides a suitable polymeric matrix because of its mechanical properties; this can result in a particulate system that efficiently performs lymphatic migration and antigen delivery to immune system cells. It has been reported that pyridyl disulfide (PDS)-functionalized star polymers have enhanced interaction with white human blood cells when compared with control star polymers. This result is interesting in the nanovaccinology field since antigen uptake by phagocytic cells, which are present in blood, is crucial for immune response activation [13]. Thiolated PAA-based particles have been designed alone or in combination with other biomaterials; however, their application has been surprisingly limited to drug delivery systems [14,15].

In this report, the synthesis and characterization of new polymeric-based particles called thiolated PAA/Schizo are described. An approach for obtaining negatively charged particles based on crosslinking thiolated poly (acrylic acid) with a *Schizochytrium* sp. cell wall fraction is described. Cytotoxicity assessment for the thiolated PAA/Schizo particles is presented and their capacity to increase the secretion of a relevant cytokine is described. Finally, the recombinant receptor binding domain (RBD) from SARS-CoV-2 was selected as the target immunogen to evaluate whether thiolated PAA/Schizo particles enhance the induced immune response to assess their potential as vehicles for vaccine development.

## 2. Materials and Methods

### 2.1. Cell Wall Fraction (CWF) Isolation

*Schizochytrium* sp. (strain ATCC 20888) was cultured at 25 °C in modified seawater medium (1 g/L yeast extract, 0.2 g/L FeSO_4_, 5 g/L peptone, 15 g/L agar, and 35 g/L NaCl). 679BY medium (1 g/L yeast extract, 1 g/L peptone, 5 g/L dextrose, and 35 g/L NaCl) was used for liquid cultures. For cell wall preparation, liquid cultures were established by inoculating a single colony in 500 mL of 679BY medium and incubating for 8 days at 28 °C and 150 rpm. The biomass was then recovered by centrifugation at 5500 rpm for 5 min and the supernatant was discarded. Cells were lysed by sonication using an ultrasound probe and the GEX130 PB device at 60% amplitude during a 10 min period comprising pulses of 30 s with 30 s of off mode in between followed by a 4 min period run at a 90% amplitude and comprising pulses of 30 s with 30 s of off mode in between. The lysate was centrifuged at 5500 rpm for 20 min at 4 °C and the supernatant was discarded. The cell wall fraction (CWF) was washed twice with 5 mL deionized water and freeze-dried (FreeZone LabConco, Kansas City, MO, USA) and then stored at 4 °C until further use.

### 2.2. Synthesis of Thiolated Polyacrylic Acid (Thiolated PAA)

Thiolated PAA was synthesized similarly to the method described previously by Greind & Bernkop-Schnürch [16]. In brief, 200 mg of PAA (450 kDa) were first hydrated in 20 mL distilled water and the pH was adjusted to 5.4 using 5 M NaOH. Afterward, 150 mM EDC was added dropwise to activate the carboxylic acid moieties of the hydrated PAA, and after a 15 min incubation under stirring at room temperature, 200 mg of L-cysteine ethyl ester were added, and pH was readjusted to 5.4 with 5 M NaOH. The reaction mixture was incubated for 3 h at room temperature under stirring. The thiolated polymer was dialyzed overnight two times against 0.2 mM HCl, two times against 0.2 mM HCl containing 1% NaCl, and finally, two times against 0.2 mM HCl. Thereafter, the pH of the conjugated result was adjusted to 6 with 5 M NaOH and frozen polymer solution was freeze-dried at −53 °C and 0.02 mbar. Dried thiolated PAA was stored at 4 °C until further use.

### 2.3. Determination of Thiol and Disulfide Groups

The amount of thiol groups immobilized on the polymer was quantified by Ellman’s method as described previously [17]. First, 0.5 mg of thiolated PAA were dissolved in 0.5 mL of 0.5 M phosphate buffer at pH 8. Afterward, 0.5 mL of Ellman’s reagent (3 mg of 5,5,’-dithiobis (2-nitrobenzoic acid) (DTNB) dissolved in 10 mL of 0.5 M buffer phosphate, pH 8) was added. Samples were incubated for 2 h at room temperature, protecting the solution from light. Samples were then transferred to a microcell and absorbance at 445 nm was measured (Cary 60 spectrophotometer). The amount of thiol groups was calculated using a standard curve (R^2^ = 0.99) prepared with solutions made to contain the unmodified polymer and cysteine ethyl ester in a 21–1.2 µg/mL concentration range. In addition, the disulfide content in the thiolated polymer was measured after reduction with NaBH_4_ and the addition of Ellman’s reagent as described by Habeeb [18]. The amount of disulfide bonds was determined by subtracting the quantity of free thiol groups from the total thiol moieties present on the polymer.

### 2.4. Determination of Amino Groups

The quantification of amino groups present in the CWF and in the thiolated PAA was performed by the ninhydrin method as previously reported [19]. Briefly, 200 mg of ninhydrin and 30 mg of hydrindantin were dissolved in 7.5 mL of DMSO. Then, 2.5 mL of 4 M acetate buffer, pH 5.2, was added and the mixture was bubbled with N_2_ for 3 min. One mL of water was added to 0.9–1.5 mg of the CWF or the thiolated PAA. Subsequently, 1 mL of the ninhydrin–hydrindantin mixture was added and samples were heated in a water bath for 20 min. Afterwards, samples were cooled on ice and 5 mL of an ethanol/water mixture (1:1) was added, followed by homogenization and absorbance measurement at 557 nm (Cary 60 spectrophotometer). The amount of amino groups was calculated using a calibration curve (R^2^ = 0.99) prepared with (3-aminopropil)trietoxisilano (APTES), which carries a single amino group per molecule, in a 1–200 μg/mL concentration range.

### 2.5. Synthesis of Thiolated PAA/Schizo Particles

CWF and thiolated PAA were used to synthesize thiolated PAA/Schizo nanoparticles by the inverse emulsion (water-in-oil (w/o)) method assisted by ultrasound. To formulate the continuous phase of the (w/o) inverse emulsion, 32.5 mL of coconut oil were mixed with 3 g of polyglycerol polyricinoleate (PGPR) (donated by Palsgaard company). An ultrasonic bath was used to sonicate the mixture for 5 min to uniformly disperse the surfactant in the oil phase. The aqueous phase solution was prepared by dissolving 12 mg of thiolated PAA in 6.6 mL of distilled water. Sixty-two mg of EDC were added to activate the carboxylic acid moieties of the thiolated PAA and the mixture was incubated for 15 min under stirring and protection from light. Afterwards, 6 mg of CWF were added to the aqueous solution. The thiolated polymer/Schizo suspension was added dropwise to the continuous phase under vigorous magnetic stirring. The emulsion was subjected to sonication at a 40% amplitude for 10 min using a high energy ultrasound probe (Sonics vibra-cell, Ultrasonic processor VCX 750 Watt); in this step, the sample heating was minimized by maintaining the sample in a cool water bath. Particles were precipitated by adding 50 mL of acetone under vigorous magnetic stirring. Finally, particles were collected by centrifugation at 12,000 rpm for 10 min and serially washed with absolute ethanol, distilled water, and 70% ethanol; the supernatant was removed in between steps. Particles were stored at 4 °C until further analysis. Thiolated PAA and non-thiolated PAA/Schizo particles were synthesized as mentioned before, omitting the CWF and using non-thiolated PAA, respectively.

### 2.6. Characterization of Thiolated PAA/Schizo Particles

#### 2.6.1. Fourier Transform Infrared Spectroscopy

The Fourier transform infrared spectra of thiolated PAA and of the PAA and cysteine ethyl ester were determined on an Agilent Cary 600 series FTIR instrument coupled with an attenuated total reflectance (ATR) accessory. Polymer thiolation was confirmed by the presence of the characteristic peaks of a newly formed amide bond. In addition, FT-IR spectra of thiolated PAA/Schizo particles and CWF were performed to analyze the functional groups.

#### 2.6.2. Hydrodynamic Diameter and Zeta Potential Measurement

The mean hydrodynamic diameter, polydispersity index (PDI), and zeta potential of freshly prepared thiolated PAA/Schizo particles and thiolated PAA plain particles were determined by dynamic light scattering (DLS) (Zetasizer Nano ZS, Malvern Instruments). Zeta potential was measured at pH 1.2, 3, 4, 5, 6, 7, 8.4, and 10. All measurements were performed at 25 °C in triplicate.

#### 2.6.3. Transmission Electron Microscopy (TEM)

Thiolated PAA/Schizo particles and *Schizochytrium* sp. CWF were analyzed by transmission electron microscopy (TEM) using a JEOL-2100 HRTEM operated at 80 kV (JEOL Ltd., Tokio, Japan). A freshly prepared sample of thiolated PAA/Schizo particles or a sample of dried CWF, resuspended by sonication in water, were placed on a copper grid, and left to dry at room temperature (CF200-Cu 200 mesh, Electron Microscopy Sciences, Hatfield, PA, USA).

#### 2.6.4. Thermogravimetric Analysis

Thermogravimetric analysis of CWF, thiolated PAA, thiolated PAA/Schizo, and thiolated PAA particles was done using a 550 TGA device (TA Instruments, New Castle, DE, USA). The thermal decomposition of the samples was carried out from 30 to 500 °C with a heating range of 10 °C/min under nitrogen gas flow.

### 2.7. In Vitro Mucoadhesiveness

The mucoadhesive strength of the thiolated PAA/Schizo particles was assessed and compared with that of non-thiolated PAA/Schizo particles by determining the percentage binding efficiency of nanoparticles to mucin. One mg of thiolated PAA/Schizo or non-thiolated PAA/Schizo particles was dispersed in 1 mL of porcine mucin (0.5 or 1 mg/mL) in simulated nasal fluid (SNF). Samples were incubated and shaken at 37 °C for 1 or 4 h. Then, suspensions were centrifuged at 10,000 rpm for 2 min. The concentration of free mucin in the supernatant was determined UV spectrometry at 260 nm (Cary 60 spectrophotometer) using a standard calibration curve prepared with mucin (18–300 μg/mL, R^2^ = 0.99). Mucoadhesiveness was expressed as mucin binding efficiency (MBE) that was calculated as follows:

$$Mucing\;binding\;efficiency\;\left(MBE\right)\%:\;\left(\frac{Total\;mucin-Free\;mucin}{Total\;mucin}\right)\times 100$$

### 2.8. In Vitro Cell Viability

The cytotoxicity of thiolated PAA/Schizo particles was evaluated by the resazurin assay in HEK 293 and murine dendritic cell lines (DC2.4 cell). HEK 293 cells were maintained in Dubelco’s modified Eagle medium (DMEM, 1X) supplemented with 10% fetal bovine serum (FBS) in a humidified incubator at 37 °C and 5% CO_2_ atmosphere. Cells were seeded in 24-well plates at a density of 5 × 104 cells per well and incubated for 24 h to allow cell attachment. DCs were cultured in RPMI medium with 10 mM HEPES supplemented with 1% nonessential amino acids, 10% fetal bovine serum, 1 mM β-mercaptoethanol, 2 mM glutamine, and 1% penicillin/streptomycin in a humidified incubator at 37 °C and 5% CO_2_ atmosphere. DCs were seeded in a 96-well plate at a density of 5 × 10^4^ cells per well.

Cell cultures were provided with thiolated PAA/Schizo particles at different concentrations (10, 100, 250, 500, and 1000 µg/mL) right after seeding and were incubated for 24 or 48 h at 37 °C in a 5% CO_2_ atmosphere. Cells given the vehicle alone (PBS) were used as a viability control, whereas cells treated with 40 mM hydrogen peroxide were used as a negative control. After incubation, the medium was replaced, and cells were incubated with 30 μg/mL resazurin for 4 h. Fluorescence was measured at 560 nm for excitation and 590 nm for emission using a FlexStation II (Molecular Devices, San Jose, CA, USA) and SoftMax Pro software Version 4.8. The supernatant from DC cultures was recovered and stored at −80 °C for subsequent cytokine analysis.

### 2.9. Cytokine Immune Assay

Cytokine secretion was evaluated by the quantification of a set of cytokines in the supernatant of DC cultures using the Cytometric Bead Array (CBA) Mouse Th1/Th2/Th17 kit (BD Bioscience), which is designed to measure the levels of IL-10, IL-17A, TNF-α, INF-γ, IL-6, IL-4, and IL-2. Briefly, cytokine standards were serially diluted to prepare calibration curves. Beads coated with seven specific capture antibodies were mixed. Subsequently, 50 μL of the mixed capture beads were blended with 50 μL of either the sample or a standard, and 50 μL of phycoerythrin (PE) and incubated for 2 h at room temperature in the dark. The samples were washed with 1 mL of wash buffer and centrifuged for 5 min. The bead pellet was resuspended in 300 μL of wash buffer after discarding the supernatant. Samples were analyzed using an Attune Thermofisher flow cytometer (Thermo Fisher Scientific, Waltham, MA, USA) and Attune NxT software v5 3.0.

### 2.10. RBD-Loaded Thiolated PAA/Schizo Particles

Recombinant SARS-CoV-2 RBD protein (YP_009724390.1, Arg319-Phe541) was purchased from Sino Biological (Cat: 40592-V08H121, purity > 95% as determined by SDS-PAGE and SEC-HPLC). The RBD protein (0.012 µg/µL) was incubated with thiolated PAA/Schizo particles (0.05 µg/µL) at 4 °C for 1 h. After incubation, samples were centrifuged at 10,000 rpm for 2 min. The concentration of free RBD in the supernatant was determined by UV absorption at 280 nm using a Thermo Scientific Multiskan FC microplate photometer (Thermo Scientific, Waltham, MA, USA) and a μDrop™ plate. A standard calibration curve was prepared using RBD (0.001–0.05 μg/µL, R^2^ = 0.96). Loading efficiency (LE) was calculated as follows:

$$Loading\;Efficiency\;\left(\%\right)=\left(\frac{Initial\;RBD-Free\;RBD}{Initial\;RBD}\right)\times 100$$

### 2.11. Immunogenicity Assessment

BALB/c mice (8–12 weeks old, *n* = 5) were randomly established and subcutaneously (s.c.) immunized with one of the following treatments: 2.5 µg RBD with 400 µg of Al(OH)_3_ (Alum) as an adjuvant (GBiosciences, St. Louis, MO, USA), 2.5 µg of RBD preincubated with 10 µg of thiolated PAA/Schizo particles, or PBS as a vehicle. Mice received three doses at 2-week intervals in a total volume of 200 µL. Blood samples were collected by tail puncture 2 weeks after final immunization. Serum was separated by centrifugation at 5000 rpm for 10 min and stored at −20 °C until further analysis. The animal study was approved by the Institutional Ethics Committee (CEID-2020-07R1).

### 2.12. Enzyme-Linked Immunosorbent Assay (ELISA)

Polystyrene plates of 96 wells were coated with RBD (80 ng/well) in a carbonate buffer with a pH of 9.6 (15 mM Na_2_CO_3_, 35 mM NaHCO_3_) and incubated overnight at 4 °C. Then, plates were washed three times with PBS; this procedure was repeated after each incubation. Plates were blocked with 5% fat-free dry milk dissolved in PBS and incubated at room temperature for 2 h. Diluted serum samples (1:300, 1:1000, and 1:2000) were added and plates were incubated for 1 h at 37 °C. Anti-mouse antibodies (IgG, IgG1, or IgG2a) conjugated with horseradish peroxidase were added as secondary antibodies and incubated for 2 h at room temperature. 2,2′-azino-bis(3-ethylbenzothiazoline-6-sulfonic acid) (ABTS, Sigma-Aldrich, MO, USA) and H_2_O_2_ were added to perform detection by measuring optical density at 405 nm (OD_405nm_) in a Thermo Scientific Multiskan FC microplate photometer (Thermo Scientific, USA).

### 2.13. Statistical Analysis

Data were presented as mean values ± standard deviation (SD) and statistical significance was performed by an analysis of variance (ANOVA) followed by Tukey’s test. Significant differences were considered for *p*-values < 0.05. Statistical analysis was performed using GraphPad Prism software version 5.01.

## 3. Results and Discussion

### 3.1. Synthesis and Characterization of Thiolated PAA

**Figure 1:**
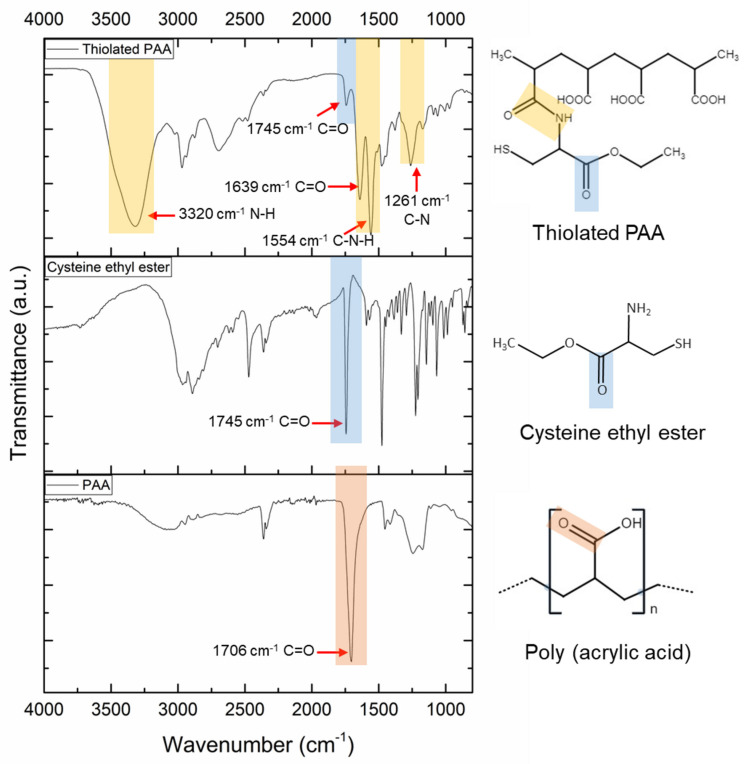
FT-IR spectra of poly (acrylic acid) (PAA), cysteine ethyl ester, and thiolated PAA.

PAA is a synthetic polymer that has been used for the synthesis of several delivery systems, mainly applied in drug release [15,20]; however, limited work has been focused on antigen delivery for further application in vaccinology. The addition of thiol groups to the polymeric chain of PAA has been an interesting strategy to increase the stability of the particle and its uptake by immune system cells [13]. In this study, thiolated PAA was synthesized using L-cysteine ethyl ester as the thiolating moiety to direct the reaction towards the carboxylic groups present in the polymer, avoiding branching. The obtained lyophilized thiolated PAA exhibited as a fibrous white powder. Amide bond formation between the NH_2_ groups of cysteine ethyl ester and carboxylic groups of PAA was mediated by EDC and confirmed by FT-IR (Figure 1). It can be noticed from the figure that the difference in the infrared spectrum between the PAA and thiolated PAA is significant. In the spectrum of PAA, the characteristic stretching absorption band of the carbonyl group C=O appears at 1706 cm^−1^ [21,22]. The cysteine ethyl ester spectrum shows a strong band at 1745 cm^−1^, corresponding to the C=O stretching of the ester group and a mild-strong band at 2550 cm^−1^ related to S-H vibration [23]. Thiolated PAA spectra present new absorption bands appearing at 3320 cm^−1^ (stretching vibrations of N-H bonds in the amide), 1745 cm^−1^ (stretching vibrations of C=O bonds of the cysteine ethyl ester molecule), 1639 cm^−1^ (stretching vibrations of C=O bonds of the amide), 1554 cm^−1^ (stretching and wagging vibrations of C-N-H bonds of the amide covalent bond formed), and 1261 cm^−1^ (stretching vibrations of C-N bonds in the amide) [20]. Therefore, the appearance of the new bands in thiolated PAA demonstrates that the cysteine ethyl ester was linked to PAA through amide bond formation via EDC.

The amount of free thiol groups and disulfide bonds distributed over thiolated PAA was quantified via Ellman’s reagent and results are presented in Table 1. About 661 μmol of thiol groups were immobilized per gram of polymer with approximately 86% remnant free thiol groups, and 14% being oxidized to disulfide bonds. The amount of free thiol groups immobilized on PAA that has been reported ranges from 90.5 to 1110 μmol/g; this wide variation could be attributed to the variation in different reaction conditions, including EDC concentration, pH value, and polymer molecular mass [24,25,26,27,28,29].

As unbound thiol is not desirable in the final material, it is imperative to remove it from the polymer prior to its use. To ensure that the dialysis purification of the conjugate was effective, in this study free cysteine ethyl ester was quantified through the ninhydrin method, in which ninhydrin and primary amino groups produce a purple dye (known as Ruhemann’s purple) with a high extinction coefficient that can be easily quantified by UV–vis spectroscopy. Purification by dialysis was deemed successful since levels of 18 ± 4 μmol of amino groups per gram polymer, which represent the unbound cysteine ethyl ester, were detected in the thiolated PAA.

### 3.2. Characterization of Thiolated PAA/Schizo Particles

Thiolated PAA/Schizo particles were prepared by inverse emulsion assisted by ultrasound, employing the previously described thiolated polymer and the CWF of *Schizochytrium* sp. microalga. It has been reported that *Schizochytrium* sp. biomass contain lipids, carbohydrates, and proteins, the latter representing up to 15% fresh weight [30]. Amino groups present in CWF were quantified by the ninhydrin method, resulting in 214 ± 42 µmol amino groups per g of CFW. Based on these results, EDC was applied to activate the remaining carboxylic groups in the thiolated PAA to promote the amide bond formation with the amine groups from the CWF.

**Figure 2:**
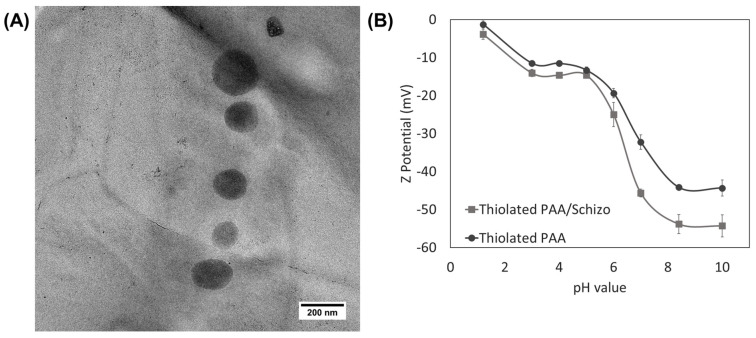
(**A**) TEM micrography of thiolated PAA/Schizo particles and (**B**) zeta potential of thiolated PAA/Schizo and thiolated PAA (plain) particles as a function of pH. Indicated values are mean values derived from experiments run in triplicate ± SD.

The resulting thiolated PAA/Schizo particles were characterized by DLS to determine their hydrodynamic diameter and zeta potential. The hydrodynamic diameter of freshly prepared thiolated PAA/Schizo particles was 313 ± 38 nm in distilled water with a PDI of 0.41 ± 0.09 and spherical morphology (Figure 2A). Thiolated PAA particles and CWF were also analyzed, showing a hydrodynamic diameter of 545 ± 73 nm with a PDI of 0.69 ± 0.06 and 442 ± 3 nm with a PDI of 0.8 ± 0.05, respectively. The incorporation of CWF in the synthesis led to particles with a smaller hydrodynamic diameter and a more homogeneous distribution. In addition, the zeta potential of thiolated PAA/Schizo particles was evaluated at different pH values and compared with that of thiolated PAA plain particles as shown in Figure 2B. Both types of particles had a negative zeta potential in the pH range evaluated, which is attributed to the presence of carboxylic groups in the PAA. However, thiolated PAA/Schizo particles showed higher stability than plain particles above pH 4. The presence of carboxylic groups from the proteins in the CWF could contribute to the negative zeta potential of thiolated PAA/Schizo particles.

**Figure 3:**
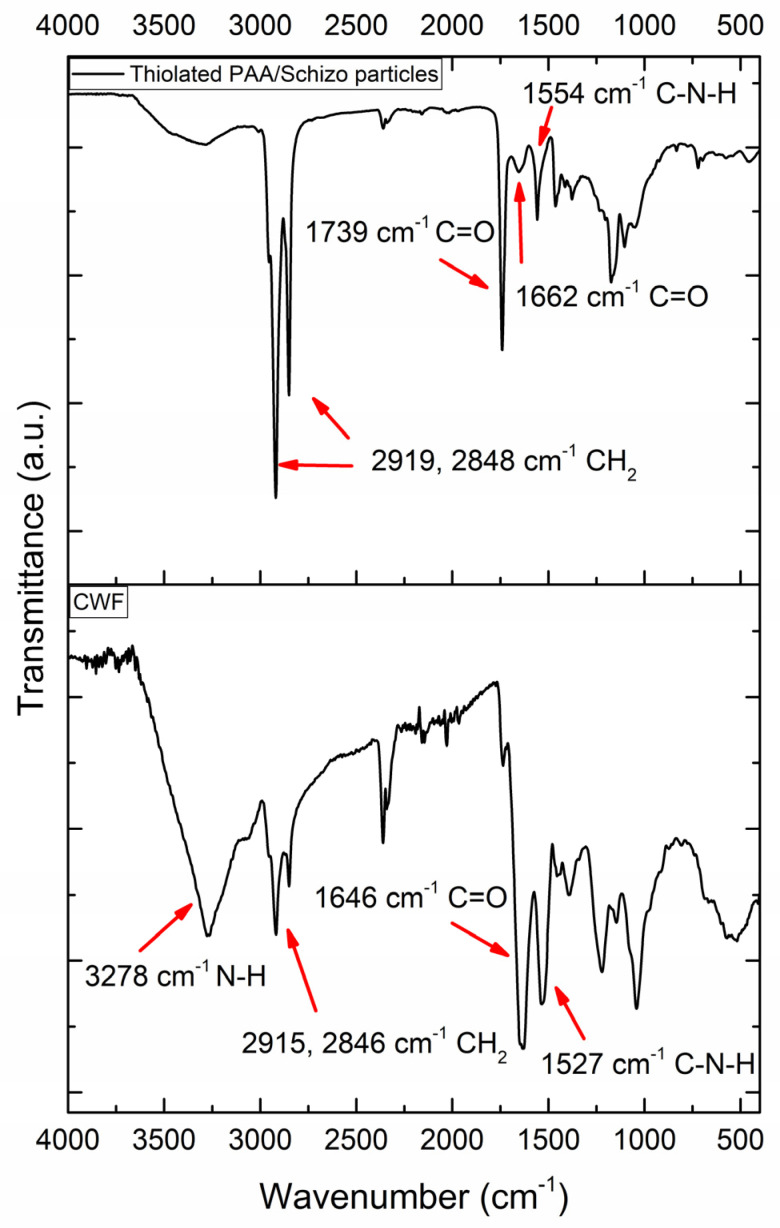
FT-IR spectra of *Schizochytrium* sp. cell wall fraction (CWF) and thiolated PAA/Schizo particles.

Thiolated PAA/Schizo particles and CWF were also characterized by FT-IR as shown in Figure 3. *Schizochytrium* sp. CWF spectra present peaks attributed to functional groups from carbohydrates, proteins, and phospholipids [31]. The band at 3278 cm^−1^ corresponds to -OH and/or -NH stretching. Symmetrical and asymmetrical -CH- vibrations of lipids can be found at 2915 and 2846 cm^−1^. The presence of proteins can be confirmed by 1646 and 1527 cm^−1^ bands which correspond to -CONH- vibrations of amide I and amide II groups. Finally, the band at 1050 cm^−1^ confirms the presence of carbohydrates. Similar bands can be observed in thiolated PAA/Schizo spectra but with different intensities. Stretching vibrations of the C=O and C-N-H of amides can be found at 1662 and 1554 cm^−1^; however, these signals can correspond to either the bond formed between cysteine ethyl ester and PAA or the proteins from CWF. The band at 1739 cm^−1^ is attributed to the stretching vibration of C=O bonds present in PAA and cysteine ethyl ester. The increase in the intensity of the bands at 2919 and 2848 cm^−1^ could be due to the presence of coconut oil residues from the synthesis. Extra purification processes were not carried out because of the natural and non-toxic origin of coconut oil. Additionally, coconut oil has been used for the synthesis of particles and has been encapsulated for different biomedical applications [32,33].

**Figure 4:**
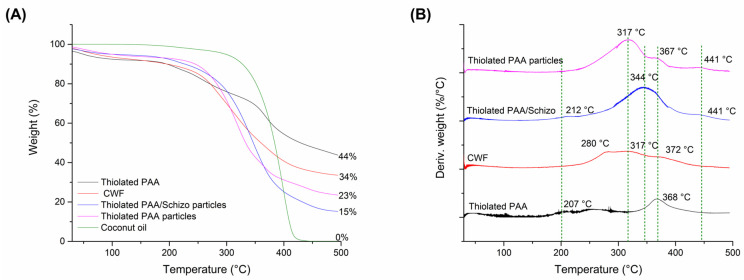
Thermogravimetric analysis (**A**) and derivative thermogravimetric (DTG) curves (**B**) of thiolated PAA, *Schizochytrium* sp. cell wall fraction (CWF), thiolated PAA particles with and without biomass, and coconut oil. Thermal decomposition of the samples was carried out from 30 to 500 °C with a heating range of 10 °C/min under nitrogen gas flow.

The thermal properties of CWF, thiolated PAA, thiolated PAA, and thiolated PAA/Schizo particles were examined by TGA. As shown in Figure 4A, the decomposition process is different in all tested samples. Thiolated PAA showed the highest thermal stability, and its decomposition process can be divided into two steps: the first one below 300 °C corresponds to the decomposition of the carboxyl groups of PAA, and the second one in the range from 340 to 400 °C is attributed to the breakage of PAA chains [34]. CWF presents initial weight loss which corresponds to the evaporation of hydrogen bond water; significant weight loss is observed in the range of 250–400 °C, which is related to the degradation of organic molecules present in the biomass [35]. Specifically, weight loss in the 250–320 °C range corresponds to the deacetylation and degradation of proteins and carbohydrates. Meanwhile, the weight loss that occurred in the 350–400 °C interval can be related to lipid content. The total percentage of the weight loss of thiolated PAA-based particles was higher than that of the thiolated PAA conjugate and CWF. In both cases, the presence of coconut oil used during the synthesis could contribute to increased weight loss.

**Figure 5:**
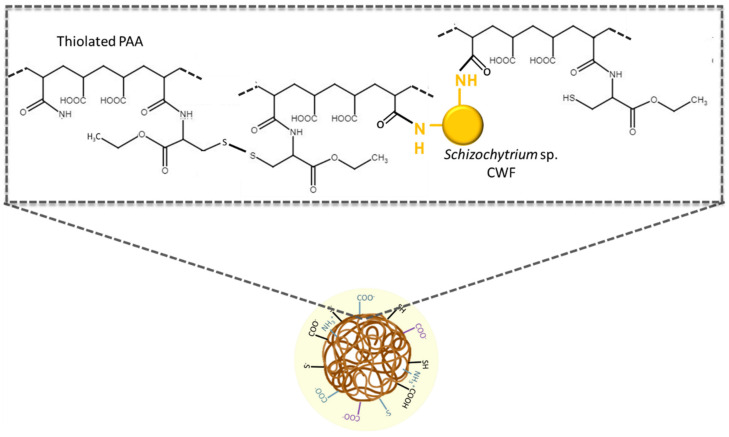
Proposed interactions in thiolated PAA/Schizo particles. Amino groups present in the cell wall fraction (CWF) of *Schizochytrium* sp. microalga can act as crosslinkers by forming amide bonds with free carboxylic groups of the PAA chain. Moreover, the presence of thiol groups can lead to the formation of disulfide bridges, which also crosslink the polymer chains.

Figure 4B shows the DTGA plots of CWF, thiolated PAA, thiolated PAA particles, and thiolated PAA/Schizo particles. The maximum degradation temperature of thiolated PAA/Schizo particles was higher compared to particles without *Schizochytrium* sp. CWF. These results indicate that the addition of CWF improves the thermal stability of the thiolated PAA-based particles. In addition, notable differences can be observed in DTGA plots in the region of 250–400 °C that are believed to correspond to the breakage of PAA chains. This shift in degradation temperatures could be due to the intermolecular interactions between the polymer and coconut oil. However, the DTGA plot of thiolated PAA/Schizo particles presents only one peak at 344 °C, which suggests strong inter- and intramolecular interactions between thiolated PAA and CWF as shown in Figure 5.

### 3.3. In Vitro Mucoadhesiveness

**Figure 6:**
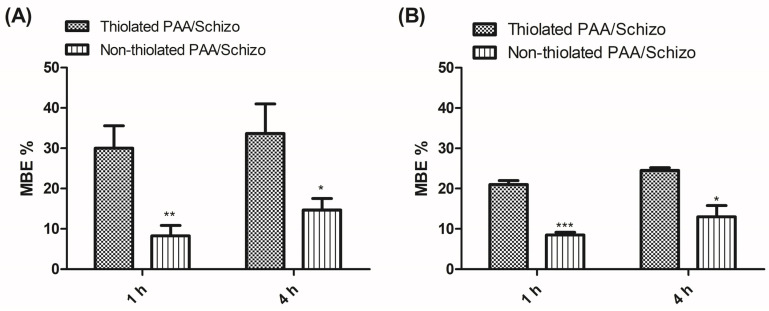
Mucoadhesion assay of thiolated and non-thiolated PAA/Schizo particles incubated at 37 °C with (**A**) 0.5 and (**B**) 1 mg/mL of mucin for 1 and 4 h. Measurements were performed in triplicate (*n* = 3 independent formulations) and indicated values are presented as means ± SD (* *p* < 0.05, ** *p* < 0.01, and *** *p* < 0.001).

Although in this study no assays were performed to determine the immunogenic properties of the thiolated PAA/Schizo particles by mucosal routes, we decided to explore its mucoadhesive properties to consider the potential to evaluate this aspect in a future study. Mucins are highly glycosylated molecules that are negatively charged at physiological pH. Since PAA is an anionic polymer, its interaction with mucins is strong at acidic pH values, when the polymer is protonated. Thus, the addition of thiol moieties is a strategy to increase polymer mucoadhesiveness. The mucoadhesive strength of thiolated PAA/Schizo particles was investigated by calculating the binding efficiency of mucin to particles. As shown in Figure 6, thiolated PAA/Schizo particles showed 30% and 34% mucoadhesion in SNF after 1 and 4 h incubation at 37 °C, respectively, whereas non-thiolated PAA/Schizo particles exhibited 8% and 15% MBE values, respectively. The bioadhesion of the thiolated polymer is higher as compared to the non-thiolated polymer due to the presence of S-S bonds in thiomers, which can covalently bond to cysteine-rich mucin subdomains [17]. These covalent bonds are stronger than the Van der Waal forces, ionic interactions, and hydrogen bonds that could be carried out in non-thiolated polymers.

### 3.4. Cytotoxicity of Thiolated PAA/Schizo Particles

**Figure 7:**
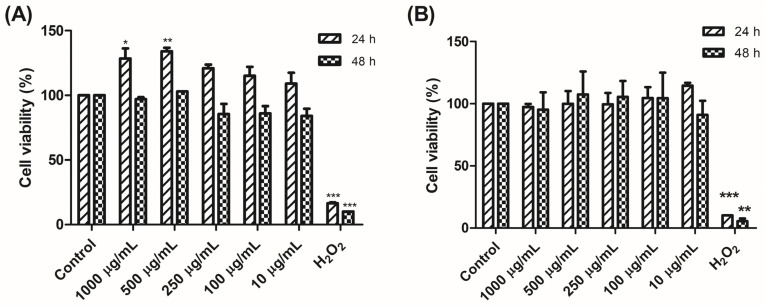
Cell viability of (**A**) HEK 293 cells and (**B**) DCs upon treatment with thiolated PAA/Schizo particles. Cells were incubated for 24 and 48 h with the test particles, the vehicle alone, or H_2_O_2_ and viability was measured by the resazurin assay. Indicated values correspond to mean values ± SD (* *p* < 0.05, ** *p* < 0.01, and *** *p* < 0.001).

The cytotoxicity of thiolated PAA/Schizo particles was evaluated by a resazurin assay in HEK 293 and DCs 4.2 cell lines in a 10–1000 µg/mL concentration range. Cell viability was not affected in the two test cell lines when exposed to concentrations up to 1 mg/mL of thiolated PAA/Schizo particles after a 24 or 48 h incubation as shown in Figure 7. Other studies have reported no harmful effects by thiolated PAA-based materials on different cell lines, including Caco-2 and human gingival fibroblast (HGF) cell lines [36,37]. Interestingly, after a 24 h incubation with thiolated PAA/Schizo particles, cell viability increased in all tested concentrations in the HEK 293 cell culture. This result is in line with reports in which coconut oil-based composites induced cell proliferation in vitro after 24 h incubation in L929 and 3T3 fibroblast cells [38,39].

### 3.5. Effect of Thiolated PAA/Schizo Particles on Cytokine Production

**Figure 8:**
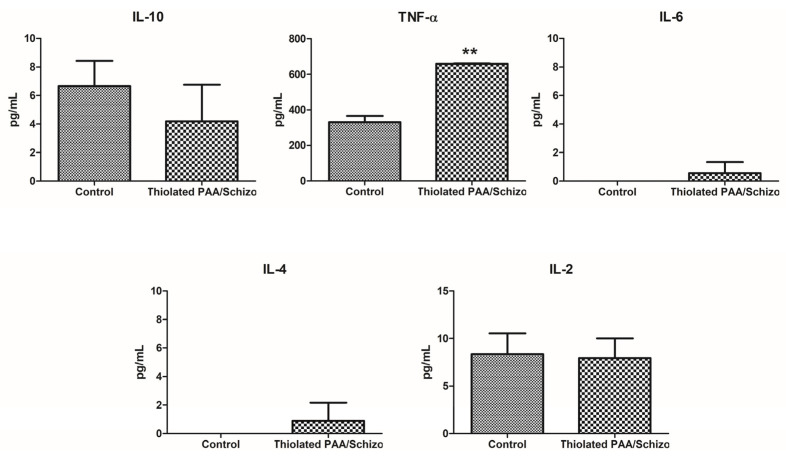
Cytokine levels in the supernatant of DCs 2.4 stimulated with 250 µg/mL of thiolated PAA/Schizo particles for 24 h (** *p* < 0.01).

DCs play an important role in presenting antigens to lymphocytes and initiating adaptative immune responses and their activation involves the increased secretion of several cytokines involved in such biological functions. Thus, the secretion of a set of relevant cytokines was determined in DCs exposed to thiolated PAA/Schizo. Figure 8 shows cytokine levels in the supernatant of DCs 2.4 stimulated for 48 h with 250 µg/mL of thiolated PAA/Schizo particles. Results showed that thiolated PAA/Schizo particles significantly induced an increased secretion of TNF-α. Moreover, low levels of IL-6 were observed in stimulated DCs, meanwhile control cells showed null detection. IL-6 and TNF-α are both cytokines involved in cellular immune responses. TNF-α is a pro-inflammatory cytokine produced by macrophages, dendritic cells, neutrophils, and lymphocytes [40], and as an effector molecule, it modulates innate immune responses by limiting the replication of infectious agents, and promoting the infiltration of macrophages, dendritic cells, natural killer cells, and neutrophils to the affected tissue area to control and clear the infection. Additionally, TNF-α is involved in cell death and it induces nuclear factor kappa B (NF-kB), which triggers the expression of proinflammatory molecules. IL-6 is produced by immune-mediated cells such as macrophages and monocytes and it exerts both anti-inflammatory and pro-inflammatory activity depending on the immune response context [41]. Pro-inflammatory activities of IL-6 include the recruitment of inflammatory cells, the inhibition of the apoptosis of inflammatory cells, and regulatory T-cell differentiation, whilst anti-inflammatory activities comprise the regeneration of epithelial cells and the induction of the hepatic acute phase response.

The production of higher levels of TNF-α cytokine after mice immunization with polymer-based nanoparticles has been observed in other studies. Li et al. [42] reported a higher level of TNF-α in both the draining lymph nodes and spleen of mice immunized with guanidyl-decorated poly(ethylene glycol)-block-poly(ε-caprolactone) nanoparticles, indicating the development of the innate immune response and antigen specific cellular immunity. Gong et al. [43] evaluated the effect of conjugated synthetic polymers (polyethylene glycol coated poly(3,4-ethylenedioxythiophene): poly(4-styrenesulfonate), PEDOT:PSS-PEG) in macrophages and DCs, observing an increase in the proinflammatory cytokines that trigger the Toll-like receptor 4 (TLR-4) pathway. Additionally, PEDOT:PSS-PEG mixed with OVA efficiently induces CD8^+^ T cell proliferation in OT-I transgenic mice and the production of cellular immunity-related cytokines.

Efforts towards the development of new adjuvants are required because only a few adjuvants or delivery systems have been licensed for human use [44]. In this field, Toll-like receptor (TLR) agonists are promising as TLR activation promotes danger signals that stimulate the innate immune system, which can lead to the evolution of adaptative immune responses. In particular, TLR-4 agonist adjuvants have been licensed for human use. The activation of TLR-4 leads to the production of proinflammatory cytokines, such as TNF-α and IL-6, which enhance the adaptive immune response through stimulating APC maturation and by inhibiting regulatory T cells and suppressing tolerance [45]. Further analysis of the cell subtypes induced by thiolated PAA/Schizo particles is required; however, the observed TNF-α levels could be an indication of their adjuvant effect.

### 3.6. RBD-Thiolated PAA/Schizo Particles Humoral Immune Assessment

RBD from the SARS-CoV-2 spike protein has been selected as a target antigen during the development of effective and safe subunit vaccines due to its interaction with the human angiotensin-converting enzyme 2 (ACE2) receptor which leads to viral cell entry [46]. Therefore, antibodies able to block RBD prevent viral entry, which explains its protective effects. The mean value for RBD loading efficiency (LE) into thiolated PAA/Schizo was 57% after 1 h incubation at 4 °C and pH 7. RBD interaction with the thiolated particles could be explained by electrostatic attraction between carboxylic groups present in the PAA and amino residues in the protein. The theoretic RBD isoelectric point is 8.9, this has a net charge of +4 at pH 7 due in part to the fact that amino residues are mostly protonated; meanwhile, carboxylic groups from thiolated PAA/Schizo particles are mainly deprotonated in this condition. Different types of nanoparticles have been widely applied as delivery systems for diagnostic and targeted therapy with the goal of maximizing cellular uptake. For such a purpose, cationic particles are preferred since they possess the ability to attach and enter cells easily due to electrostatic attraction to the negatively charged cell membrane glycoproteins. Nevertheless, reports have suggested that positively charged carriers can induce toxicity as they are more likely to disrupt membrane integrity and strongly bind to the negatively charged DNA, causing its damage [47]. The negative zeta potential profile shown by thiolated PAA/Schizo particles could be unsuitable for inducing a highly favorable interaction with cell membranes; however, these properties allowed the efficient absorption of a positively-charged antigen without requiring chemical conjugation.

**Figure 9:**
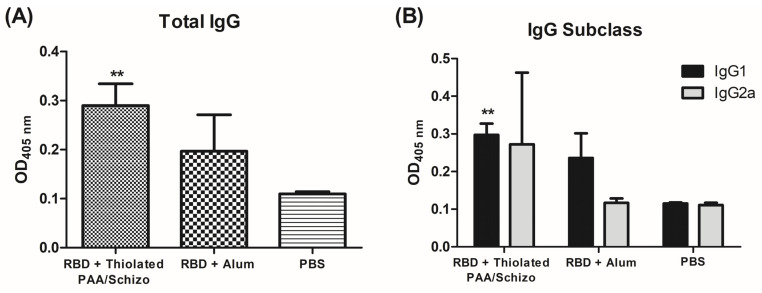
Humoral immune response induced by 2.5 µg RBD plus thiolated-PAA/Schizo or alum in test mice. (**A**) Antigen-specific total IgG and (**B**) IgG subclass antibody levels in sera after three s.c. immunizations. Serum levels of total IgG were determined by ELISA at a 1:1000 dilution; IgG1 and IgG2a were determined at 1:300 dilution. Data are presented as mean values ± standard deviation (SD) and statistical significance was determined by analysis of variance (ANOVA) followed by Tukey’s test. Significant differences are indicated as, ** *p* < 0.01 (versus immunized mice with RBD plus thiolated PAA/Schizo particles).

The subcutaneous route was selected to screen the immunogenicity of the obtained thiolated PAA/Schizo particles, as the primary approach to determine the feasibility to use this material for vaccine delivery. Since mucoadhesiveness was deemed promising, a future study will be focused on determining the immunogenicity by mucosal routes. Thus, BALB/c mice were immunized with recombinant RBD protein and thiolated PAA/Schizo particles to analyze the induced humoral immune response compared with the use of alum as a conventional adjuvant used as a reference. BALB/c mice were s.c. immunized with 2.5 µg RBD preincubated with thiolated PAA/Schizo particles and the humoral immune response was measured and compared with that induced by the same RBD dose with alum as an adjuvant. As observed in Figure 9A, mice vaccinated with RBD plus thiolated PAA/Schizo particles showed higher total IgG response in serum diluted 1:1000 after receiving the third dose compared to mice immunized with RBD plus alum; however, the mean value of anti-RBD total IgG titers was 2000 for both test groups. The humoral response was further analyzed in terms of the relative levels of antigen-specific IgG1 and IgG2a subclasses. As shown in Figure 9B, RBD adjuvanted with alum induced higher levels of IgG2a compared to IgG1 levels. In contrast, RBD-thiolated particles induced a more Th1/Th2 balanced response since IgG2a and IgG1 levels were statistically indistinguishable.

Subunit-based formulations stand out as advantageous vaccines due to their defined composition, safety, and convenient production; however, this type of vaccine tends to be less immunogenic compared to whole pathogen-based vaccines, and thus requires the use of adjuvants to achieve a proper potency to achieve immunoprotection. Several adjuvants have been used in approved and experimental vaccines; however, this field requires an expansion of the adjuvants available for human use to cover the demand and improve some aspects that include safety, cost, and immunomodulation [48]. Thus, the development of new adjuvants is a relevant and often challenging goal. The immune system comprises innate and adaptive immune responses against harmful or foreign agents [49]. In general, innate immunity is considered the body’s first line of defense against pathogens; it is considered a quick non-specific immune response. Adaptive immunity responds to specific antigens from pathogens and involves cellular-mediated (Th1) and humoral-mediated (Th2) immune responses [50]. The Th1 immune response offers protective immunity against intracellular infectious agents and enhances the production of IgG2a antibodies in mice, while the Th2 immune response is involved in host defense against extracellular pathogens and promotes the production of IgG1 antibodies. Th1/Th2 balanced immune response relies on a collective effort of several immune cell subsets to achieve an appropriate defense response against harmful agents [51].

In this study, alum was used to compare the potential adjuvant effect of thiolated PAA/Schizo particles. Alum adjuvants have been widely used in vaccines approved for humans [48]. Their mechanisms of action include the induction of local inflammation, depot effect, and promotion of Th2-based antibody response with minimum or no Th1 immune responses. Interestingly, thiolated PAA/Schizo particles induced higher levels of both IgG1 and IgG2a antibodies in mice, suggesting a balanced Th1/Th2 response. Other polymer-based particles have shown an induction of balanced Th1/Th2 responses. Ovalbumin-loaded chitosan nanoparticles induced specific IgG, IgG1, and IgG2a antibody responses and promoted the production of Th1 and Th2 cytokines [52]. Oil-based adjuvants have also been used in licensed and experimental vaccine candidates. MF59 is an adjuvant licensed for humans that contains oil (squalene) and is able to boost both Th1 and Th2 immune responses. Hybrid polymeric–lipid particles have also been used for nanovaccine formulations. Hyaluronic acid (HA)-decorated cationic lipid–poly(lactide-co-glycolide) acid (PLGA) hybrid nanoparticles loaded with ovalbumin induced a balanced Th1/Th2 immune response [53]. *Schizochytrium* sp. has been used not only as a host for recombinant antigen production but also as the delivery vehicle given that it contains immunostimulatory compounds, such as palmitic acid, squalene, and polysaccharides, that could enhance the induced immune response [7,54]. Moreover, coconut oil residues in thiolated PAA/Schizo particles could be involved in the induction of humoral and cellular immune responses; however, a broader study of different immune cell subsets and cytokine production must be carried out to ascertain this point.

## 4. Conclusions

This paper showed the viability of synthesizing thiolated PAA-based particles crosslinked with *Schizochytrium* sp. CWF synthesized by emulsion using coconut oil as the natural lipid phase. PAA chemical modification carried out through carboxylic group activation allowed the generation of amide bonds with cysteine ethyl ester molecules which provided thiol groups with the objective of increased stability and uptake by immune system cells. Thiolated PAA was successfully crosslinked with the cell wall fraction through new amide bond formation between the remaining carboxylic groups from PAA and amine groups from CWF proteins. TGA analysis suggested intermolecular interactions between the thiolated polymer, CWF, and remaining coconut oil. The thiolated PAA/Schizo particles that were formed showed a spherical shape with a 313 ± 38 nm hydrodynamic diameter, negative zeta potential, and increased mucoadhesiveness and showed no toxic effect in vitro. Moreover, thiolated PAA/Schizo particles significantly induced an increased secretion of TNF-α by DCs which could be an initial indication of its adjuvant effect, which was confirmed by immunization assays in which thiolated PAA/Schizo particles aided in the induction of humoral responses that were similar in magnitude to those induced by alum but with a more balanced IgG2a/IgG1 ratio, suggesting that this carrier acts as an adjuvant capable of inducing balanced cellular and humoral responses against intracellular pathogens. Based on the mucoadhesive properties of thiolated PAA/Schizo particles, there is a potential to assess this system in mucosal-based immunization schemes.

## Figures and Tables

**Table 1:** Levels of thiol groups and disulfide bonds immobilized in thiolated PAA as well as number of amino groups associated with the remaining unbound cysteine ethyl ester in the conjugated result after dialysis and lyophilization. Indicated values are mean values derived from experiments run in triplicate ± SD.

Sample	-SH (μmol/g)	S-S (μmol/g)	Ʃ-SH (μmol/g)	-NH_2_ (μmol/g)
Thiolated PAA	569 ± 56	46	661 ± 24	18 ± 4

## Data Availability

The original contributions presented in the study are included in the article, further inquiries can be directed to the corresponding author/s.
